# Prevalence of common mental disorders and associated factors among adults with Glaucoma attending University of Gondar comprehensive specialized hospital tertiary eye care and training center, Northwest, Ethiopia 2020

**DOI:** 10.1371/journal.pone.0252064

**Published:** 2021-05-20

**Authors:** Mikias Mered Tilahun, Betelhem Temesgen Yibekal, Habtamu Kerebih, Fisseha Ademassu Ayele

**Affiliations:** 1 Department of Optometry, School of Medicine, College of Medicine and Health Science, University of Gondar Comprehensive specialized hospital, Gondar Town, Ethiopia; 2 Department of Psychiatry, School of Medicine, College of Medicine and Health Science, University of Gondar Comprehensive Specialized Hospital, Gondar Town, Ethiopia; 3 Department of Ophthalmology, School of Medicine, College of Medicine and Health Science, University of Gondar Comprehensive Specialized Hospital, Gondar Town, Ethiopia; St. Paul’s Hospital Millenium Medical College, ETHIOPIA

## Abstract

**Purpose:**

This study aimed to assess the prevalence of common mental disorders and associated factors among adults with glaucoma at Gondar university comprehensive specialized hospital tertiary eye care and training center. Glaucoma predisposes patients to common mental problems and leads to wasteful, costly and inefficient use of medical services and complications of the diagnoses. So, determining the level and factors associated with common mental disorders among glaucoma patient would help to improve and integrate comprehensive ophthalmic services which address common mental disorder in a follow-up visit.

**Methods:**

An institution-based cross-sectional study was conducted on 495 glaucoma patients selected by using systematic random sampling. Data were collected through face-to-face interview and chart review. Self-reported questionnaire (SRQ-20) was used to assess the presence of common mental disorders. Binary logistic regression analysis was done to identify factors associated with common mental disorders. Variables with P<0.05 were considered as factors significantly associated with common mental disorders.

**Result:**

Four hundred sixty-eight patients were included in this study with a response rate of 94.54%. The mean age of the participant was 58 ± 14.11 years. The prevalence of common mental disorders was found to be 29.5% (95% CI 25.4–33.3). Female sex (AOR = 3.79, 95% CI: 1.66–8.62) (p-value = 0.001), average monthly income of less than 1200 birr (AOR = 6.05 95% CI: 2.26–16.22**) (**p-value **=** 0.001), poor level of social support (AOR = 17.39 95% CI: 7.79–38.82) (p-value = 0.001), moderate and high risk of alcohol use (AOR = 10.42 95%CI: 2.74–39.54) (p-value = 0.001), presence of chronic medical illness (AOR = 3.85 95% CI: 2.07–7.16) (p-value = 0.001), receiving both drug and surgical treatment (AOR = 2.50, 95%CI: 1.30–4.83) (p-value = 0.006) and presence of systemic carbonic anhydrase inhibitors use (AOR = 3.16, 95%CI: 1.65–6.06) (p-value = 0.001) were significantly associated with increased level of common mental disorders.

**Conclusion:**

Significant numbers of glaucoma patients have CMD and found significantly associated with socio-economic, ocular and systemic clinical factors. Therefore, the integration of psychosocial care into the current treatment of patients with glaucoma would have a significant advantage to help these patients.

## Introduction

Glaucoma is a chronic, progressive, heterogeneous optic neuropathy, which leads to visual field loss, disabilities, and irreversible blindness [1]. Glaucoma is the second cause of irreversible blindness in the world with a prevalence of 3.54% between the populations of 40–80 years of age [2].

Africa takes the most prevalence of primary open-angle glaucoma in the world (4.20%) [2]. The disease is also one of the leading causes of blindness and visual impairment in Ethiopia [3].

Common mental disorders are a group of distress states manifesting with anxiety, depression, and unexplained somatic symptoms. They usually manifest with shifting combination of symptoms over time indicating emotional or mental abnormality [4, 5].

Several studies conducted across the world reported the prevalence of common mental disorders among glaucoma patients ranging from 18%-36% [6, 7]. A study done in Ethiopia revealed that 23.2% of adult glaucoma patients had CMD [8]. According to WHO’s report in 2011, CMDs are predicted to be the first leading cause of disease burden by the year 2030 [5]. They have been recognized as a common co-morbid illness with other medical conditions, particularly the disease which has chronic nature [4].

Progressive vision loss, lifelong application of multiple medications, frequent follow-ups, expenses related with those issues and productive loss due to visual loss lead glaucoma patients to comorbid common mental disorders (CMDs) [9–12]. Moreover, the studies also revealed that a chemical change initiated by the disease can have the ability to cause mental problems [13]. Vision loss is one of the leading causes of disability and is associated with reduced quality of life and increased depressive and anxiety symptoms [14, 15]. Depression and anxiety may cause a further decline in quality of life, may aggravate disability caused by the visual impairment and may increase vulnerability for health decline [16].

Depression and anxiety constitute a greater percentage of these co-morbid psychiatric disorders in glaucoma patients, which are often not considered by eye care practitioners who commonly emphasize on ophthalmic care of their patient [10, 13, 17–19].

Neglected comorbid mental problems impose an extra burden on the patient who struggles from the visual problem of the disease [11]. So those untreated and disregard comorbid psychiatric illnesses lead to worsening of the condition, a longer hospital stay, and increased costs of care [20]. This often leads to wasteful, costly, and inefficient use of medical services and complications of the diagnosis [7, 21, 22].

There is limited information about common mental disorders among glaucoma patients in Ethiopia in general and in the study area in particular. So this study will be used as reference for further studies and be of assistance in assessing the psycho-social burden of the disease which helps to tackle these comorbidities which vantage to decrease the quality of life of the patient. So this study aimed to assess the prevalence of the common mental disorder among adults with glaucoma and factors contributing to its future integrated and comprehensive intervention.

## Methods

### Study design and period

An institutional-based cross-sectional study conducted from June to August 2020.

### Study area

The study was conducted at University of Gondar comprehensive specialized hospital tertiary eye care and training center in Gondar town, Ethiopia. This tertiary eye care is divided into 8 subspecialty clinics, which are anterior segment, Glaucoma, Retina, Pediatrics and strabismus, oculoplasty, Refraction, binocular vision, and low vision. Glaucoma clinic is a major subspecialty which provides care for about 960 glaucoma follow-up patient in a month.

### Source population

All adult glaucoma patients (age ≥18 years) who had glaucoma follow-up at the University of Gondar comprehensive specialized hospital tertiary eye care and training center.

### Study population

All adult glaucoma patients (≥18 years) who had glaucoma follow-up at the University of Gondar comprehensive specialized hospital tertiary eye care and training center who came during the data collection period were included. Glaucoma patients linked to the clinic for less than one month, prior clinical diagnosis of patients with depression or anxiety, and patients unable to answer the questionnaire due to general disability were excluded.

### Sample size determination

This study had two specific objectives which were to estimate the prevalence of common mental disorders among adult glaucoma patients and to identify factors associated with common mental disorders. So sample size was calculated for the two objectives separately. The sample size for the first objective was calculated by considering the prevalence of common mental disorders among adult glaucoma patients as 23.2%(which is taken from a similar study done in Addis Ababa, Ethiopia(8) so P = 0.23 and q = 0.77

$$Sample\;size\;\left(n\right)=\frac{{\left(Z_{\alpha /2}\right)}^{2}\;x\;p(1‐p)}{d^{2}}=\frac{\left(1.96)2\;x\;0.23(1‐0.23\right)}{{\left(0.04\right)}^{2}}=428$$

Where n = estimated sample size, p = prevalence of common mental disorder, Z_α/2_ = value (Z-statistic) at the 95% confidence level (α = 0.05) which is 1.96, d = margin of error 4% (0.04).

The sample size for objective two was calculated by considering factors that shows a consistent significant association with a common mental disorder in previous studies. By taking a similar study done in Addis Ababa Ethiopia, gender, monthly income, and duration of illness were significantly associated factors with common mental disorders [8]. By using open epi computer software and considering 95% CI, 80% power the sample size calculated considering the duration of illness was larger. It was calculated by considering the ratio of patients without CMD to patients with CMD 4:1, 9.1% unexposed with the outcome, 14% exposed with the outcome, and odds ratio of 2.01 which gives the generated sample size of 450.

Since the calculated sample size for objective two was larger it was used to determine the final sample size. After considering a 10% non-response rate the final sample size becomes 495.

### Sampling procedure

A systematic random sampling method was used to select the study participants. The glaucoma clinic gives service to patients on Monday, Wednesday, and Friday. From the available information, the glaucoma patients attending the center are 120 per week, which are about 40 in each day. The patients follow-up registration book was used as a sampling frame. Among these patients, the sampling fraction K was calculated using the total number of glaucoma patients in two months = 960 (so K = 960/495 = 2). The first patient was selected by using the lottery method then the next patient was selected at 2 intervals on each day within the study period.

### Data collection tool and procedure

A structured interviewer-administered questionnaire was used to collect the data. It has questions on socio-demographic characteristics, symptoms of common mental disorders, ocular health status, systemic health conditions, level of social support and substance use. Except for questions on ocular health status which was collected by chart review other questions we translated to the local language Amharic. Self-reported questionnaire (SRQ-20), which was developed by the WHO was used to assess the presence of common mental disorders. The SRQ was originally designed as a self-administered scale, but was also found to be suitable for interviewer-administered questionnaires because of the low literacy rate in developing countries [23]. It was also translated Amharic language and validated for use in Addis Ababa Ethiopia [24]. The instrument performed well in detecting common mental disorders, with an area under the curve (AUC) of 0.879 (SE  =  0.23, 95% CI 0.83–0.92) to the overall sample and with an optimal cut-off score at 5/6 with a sensitivity of 78.6% and specificity 81.5%. Each of the 20 items is scored 0 or 1. A score of 1 indicates that the symptom presents during the past month, a score of 0 indicates that the symptoms absent. A cutoff point of 11 and above was considered as having CMD. It is most commonly used in developing countries and also in Ethiopia among patients in health care settings [25].

Social support was measured by the Oslo-3 scale as lower, moderate, and strong levels of social support. Its internal consistency could be regarded as acceptable with *α*  =  0.640 [26].

Substance use was measured by Alcohol, Smoking, and Substance Involvement Screening Test (ASSIST) which was developed by WHO. The ASSIST accurately identified tobacco, alcohol, and cannabis use disorders (sensitivities = 95%-100%; specificities = 79%-93%; area under the curve [AUC] = 0.90–0.94). It determines a risk score for each substance which ranges from 0–27 then the score obtained for each substance falls into a lower, moderate or high risk category [27].

The questionnaire also included an additional question assessing patients’ general health status. A chart review was done to get information on the ocular health related factors. The interview was done by 4 trained optometrists.

### Operational definitions

#### Common mental disorders

A score of eleven or more on the SRQ-20 in the past four weeks was considered as having common mental disorders [23].

#### Level of social support

Support at a time when difficulties and critical conditions like financial, social, and psychological problems arise. Based on the Oslo 3 scale Poor support: 3–8, moderate support: 9–11, and Strong support: 12–14 [28].

#### Substance use

Using the Alcohol, Smoking, and substance involvement screening test (ASSIST) lower risk 0–10, moderate risk 11–26, and high risk 27+ [27].

### Data processing and analysis

The collected data was entered into Epi-info version 7 and exported to SPSS version 20 for analysis. Summary statistics, frequencies, and cross-tabulations were performed for the descriptive data. Binary logistic regression was conducted to identify factors associated with CMD. The model fitness was checked using the Hosmer and Lemeshow model fitness test. An adjusted odds ratio with 95% confidence interval was used to measure the strength of association between dependent & independent variables. Variables with a p-value less than 0.05 were considered statistically significant.

### Ethical consideration

The study was conducted as per the Declaration of Helsinki and approved by the University of Gondar Ethical Review Board. Ethical clearance was obtained from the institutional review board of the University of Gondar, college of medicine and health science, school of medicine ethical review committee. A permission letter was also obtained from the hospital administration and ophthalmology department. An informed verbal consent was obtained from each participant after giving a clear explanation about the purpose of the research.

Study participants were assured their response will be kept confidential and no personal identifiers were used. During the data collection 32 patients who had relatively higher CMD scores and ASSIST score were linked to the hospital’s psychiatry clinic for further psychiatric evaluation.

## Results

### Socio-demographic and economic characteristics of the study participants

Four hundred sixty-eight adult glaucoma patients participated in the study with a response rate of 94.54%. The mean age of the participants was 58 ± 14.11 years. Majority of them were male (65%). Married participants account 77.2% of the respondents. Around 44.7% of the participants had an average family income of greater than 1200 ETB per month. (Table 1).

**Table 1:** Socio-demographic and economic characteristics of study participants.

Characteristic	Frequency	Percent
Sex		
Male	304	65
Female	164	35
Age		
18–39	45	9.6
40–59	181	38.7
>60	242	51.7
Marital status		
Married	362	77.4
Single	23	4.8
Divorced	28	6
Widowed	55	11.8
Educational status		
Cannot read and write	131	28
Read and Write	128	27.4
Primary school	55	11.8
Secondary school	62	13.2
Collage and Above	92	19.6
Religion		
Orthodox	389	83.1
Muslim	74	15.8
Others	5	1.1
Occupation		
Farmer	158	33.7
Housewife	109	23.3
Self-employee	95	20.3
Government employee	63	13.5
Retired	43	9.2
Residence		
Rural	239	51.1
Urban	229	48.9
Family average income		
<750 ETB	143	30.8
750–1200 ETB	116	24.5
>1200 ETB	209	44.7
Family size		
1–3	26	5.6
4–6	361	77.1
7–10	81	17.3

### Level of social support, substance use, and comorbid chronic medical illness among study participants

Among the study participant, 53.6% had a poor level of social support, 73.5% had lower risk of alcohol use and 19.2% of them had Diabetes Mellitus. (Table 2). All individuals who participated in this study had a lower risk of Tobacco and Amphetamines type of stimulants use.

**Table 2:** Level of social support, substance use and comorbid medical illness among study participants.

Characteristic	Frequency	Percent
Level of Social support		
Poor	251	53.6
Moderate	217	46.4
Alcoholic beverages		
Lower Risk	344	73.5
Moderate Risk	102	21.8
High Risk	22	4.7
Chronic medical illness		
Diabetes Mellitus	90	19.2
Hypertension	23	4.9
Both DM and HTN	22	4.7
Others	22	4.7
No chronic illness	311	66.5
Tobacco use		
Lower Risk	468	100
Amphetamine use		
Lower Risk	468	100

### Ocular clinical characteristics of study participants

Regarding the ocular clinical characteristics, the most prevalent type of glaucoma was primary open-angle glaucoma 209 (44.6%) followed by Pseudoexfoliative glaucoma 159 (34%). Most of them had moderate glaucoma 216 (46.2%) and the majority of the patients had bilateral glaucoma 376 (80.3%). (Table 3).

**Table 3:** Ocular clinical characteristics of study participants.

Characteristics	Frequency	Percent
Visual acuity		
No visual impairment	203	43.4
Moderate visual impairment	214	45.7
Blindness	51	10.9
IOP in mmHg		
<18	63	13.5
18–30	364	77.7
>30	41	8.8
Cup to disc ratio		
< 0.65	36	7.7
0.7–0.9	216	46.2
0.9–1	216	46.2
Type of Glaucoma		
Primary Open Angle Glaucoma	209	44.6
Pseudoexfoliative Glaucoma	159	34
Other secondary open-angle glaucoma	78	16.7
Angle-closure Glaucoma	22	4.7
Severity of Glaucoma		
Mild	36	7.7
Moderate	216	46.2
Advanced	174	37.2
Absolute	42	9.0
Laterality of Disease		
Bilateral	376	80.3
Unilateral	92	19.7
Ocular comorbid condition		
Yes	316	67.5
No	152	32.5

### Type and duration of glaucoma treatment among study participants

Most of the study participants 378(80.8%) had only medication therapy and the majority of them 422(90.2%) had monotherapy. From those who had surgical treatment majority of them had trabeculectomy. (Table 4).

**Table 4:** Type and duration of glaucoma treatment among study participants.

Type of Treatment		
Drugs only	378	80.8
Drug and Surgery	90	19.2
Types of Glaucoma surgery		
Trabeculectomy	78	16.6
Laser PI	12	2.6
Number of Eye Drops		
One	422	90.2
More than one	46	9.8
Systemic carbonic anhydrase inhibitors		
Yes	120	25.6
No	348	74.4
Duration of treatment		
≤12 month	216	46.2
> 12 month	252	53.8
Past eye surgery		
Yes	117	25
No	351	75

### The prevalence of common mental disorders

The prevalence of CMD among glaucoma patients was 29.5% (95%CI 25.4–33.3).

### Factors associated with common mental disorders among study participants

The result of multivariable logistic regression showed that gender, monthly income, level of social support, alcohol use, type of treatment, systemic carbonic anhydrase inhibitors use, and presence of systemic comorbidity had a significant association with common mental disorders. (Table 5).

**Table 5:** Factors associated with common mental disorders among study participants.

Characteristics	CMD		COR (95%)	AOR (95%)	p-value
	Yes	No			
Sex					0.001
Male	76	228	1	1	
Female	62	102	1.82(1.21–2.74)	3.79(1.66–8.62)*	
Occupation					
Farmer	52	106	2.60(1.22–5.52)	0.55(0.13–2.36)	0.42
House Wife	38	71	2.84(1.29–6.2)	0.59(0.14–2.35)	0.45
Self employee	22	73	1.59(0.66–3.6)	1.39(0.43–4.51)	0.58
Retired	16	27	3.14(1.25–7.89)	0.37(0.08–1.67)	0.19
Government	10	53	1	1	-
Residence					0.12
Rural	81	158	0.64(0.43–0.96)	0.15(0.05–0.41)	
Urban	57	172	1	1	
Family average income					
< 750 ETB	65	78	3.52(2.19–5.67)	6.05(2.26–16.22)**	0.001
750–1200 ETB	33	83	1.68(0.99–2.85)	3.74(1.44–9.70)	0.007
>1200 ETB	40	169	1	1	-
Level of social support					0.001
Poor	121	130	10.95(6.29–19.04)	17.39(7.79–38.82)**	
Moderate	17	200	1	1	
Alcoholic beverage use					
Lower risk	85	259	1	1	-
Moderate risk	38	64	1.80(1.13–2.89)	1.97(0.93–4.19)	0.075
High risk	15	7	6.52(2.57–16.55)	10.42(2.74–39.54)**	0.001
Visual Acuity					
No visual impairment	44	159	1	1	-
Moderate visual impairment	54	117	2.33(1.21–4.48)	0.97(0.38–2.46)	0.95
Severe visual impairment	20	23	3.14(1.58–6.24)	1.72(0.65–4.59)	0.27
Blindness	20	31	1.66(1.04–2.65)	0.93(0.47–1.86)	0.85
IOP in mmHg					
<18	16	47	1	1	-
18–30	104	260	1.17(0.64–2.16)	1.90(0.80–4.53)	0.14
>30	18	23	2.29(0.99–5.31)	1.33(0.39–4.45)	0.64
Type of Glaucoma					
Primary open-angle glaucoma	48	161	1	1	-
Pseudoexfoliative glaucoma	57	102	1.87(1.18–2.96)	1.56(0.80–3.03)	0.19
Other secondary open-angle glaucoma	25	53	1.58(0.98–2.81)	0.79(0.35–1.81)	0.58
Angle-closure glaucoma	8	14	1.91(0.76–4.84)	0.88(0.18–4.21)	0.87
Severity of Glaucoma					
Mild	10	26	1	1	-
Moderate	50	166	0.78(0.35–1.73)	0.43(0.14–1.28)	0.13
Advanced	62	112	1.44(0.65–3.18)	0.69(0.23–2.05)	0.50
Absolute	16	26	1.60(0.61–4.17)	0.33(0.08–1.33)	0.12
Type of Treatment					0.006
Drugs only	92	286	1	1	
Drugs and Surgery	46	44	3.25(2.02–5.22)	2.50(1.30–4.83)*	
Number of eye drops					0.57
One	112	310	1	1	
More than one	26	20	3.99(2.27–6.99)	1.28(0.54–3.04)	
Systemic carbonic anhydrase inhibitors					0.001
Yes	65	55	4.45(2.86–6.92)	3.16(1.65–6.06)**	
No	73	275	1	1	
Duration of Treatment					0.99
≤12 month	53	163	0.64(0.43–0.96)	1.00(0.55–1.80)	
>12 month	85	167	1	1	
Ocular Co-morbid condition					0.62
Yes	102	214	1.53(0.99–2.39)	0.84(0.41–1.71)	
No	36	116	1	1	
Past Eye Surgery					0.52
Yes	42	75	1.48(0.95–2.32)	0.80(0.41–1.58)	
No	96	255	1	1	
Chronic medical illness					0.001
Yes	60	251	4.13(2.71–6.29)	3.85(2.07–7.16)**	
No	78	79	1	1	

**P<0.01

*p<0.05.

The likelihood of CMDs was about four-fold higher among women as compared to men (AOR = 3.79 95%CI: 1.66–8.62) (p-value = 0.001). Subjects who had <750ETB monthly income were six times more likely to have CMD compared to those who had >1200ETB per month (AOR = 6.05 95%CI: 2.26–16.22**)** (p-value = 0.001).

The likelihood of common mental disorders among study participants with poor level of social support was seventeen times higher than participants with moderate level of social support (AOR = 17.39 95%CI: 7.79–38.82) (p-value = 0.001). A subject with high risk of alcohol use were ten times more likely to have CMD than participants with low risk of alcoholic beverage user (AOR = 10.42 95%CI: 2.74–39.54) (p-value = 0.001).

Respondents who were on drugs and surgical treatment were about three times more likely to experience CMDs compared to those who were only on drug therapy (AOR = 2.50, 95%CI: 1.30–4.83) (p-value = 0.006). Study participants on systemic carbonic anhydrase inhibitors were three times more likely to develop CMD compared with a client without systemic carbonic anhydrase inhibitors (AOR = 3.16, 95%CI: 1.65–6.06) (p-value = 0.001).

Participants with chronic medical illness were more than three times more likely to have CMDs compared with those without chronic illness (AOR = 3.85 95%CI: 2.07–7.16) (p-value = 0.001).

## Discussion

This study assessed common mental disorders using SRQ-20 among glaucoma patients. Common mental disorders were observed in 138 (29.5% (95% CI 25.4–33.3) participants. This was lower than the study done in Nigeria [7], which concluded 36.9% of glaucoma patients had comorbid CMD. This might be due to the difference in sample size which is larger in the current study, and difference in population characteristic. A relatively higher prevalence of CMD was observed in the current study than reports from Egypt (18.3%), Nigeria (22%), and Addis Ababa, Ethiopia (23.2%) [6, 8, 29]. The possible reason for this could be difference in study design, and study setting. For instance, the study in Ethiopia include only 423 individuals, about (83%) of the study participant came from urban which is higher than (48.9%) in the current study, (69%) had >1200 ETB monthly income which is higher than current study where only (44.7%) and population characteristic difference.

Women were about four times more likely to experience CMD compared with men. This, finding is consistent with studies done in Ethiopia [8], Egypt [6], Turkey [11], and USA [10]. It has been shown that mental health problems particularly depression, anxiety, and somatic complaints affect women to a greater extent than men across diverse societies and social contexts [30]. The reason might be women are responsible for most of the household chores and child education [31], in addition to performing functions resulting from their inclusion in the formal job market, thus causing a work overload in the female population. The excess of attributions can create conflicting situations, stress and suffering and also be associated with greater psychiatric morbidity [31, 32]. Low income had a significant association with common mental disorders. This result is in line with studies done in Ethiopia [8] and China [33]. This might be due to the fact that having low-income leads to lack of financial resources to afford the frequent care needed in glaucoma treatment alongside fulfilling basic needs to maintain basic living standards. In addition, since the economic burden of glaucoma is substantial and increases as the disease progresses [34] this might predispose patients to CMD.

The likelihood of developing common mental disorders among those with poor level of social support was seventeen folds higher as compared to a moderate level of social support. This finding is in agreement with a study done in Nigeria [7]. The reason for this could be the ability of good social support to protect people from CMD through emotional support and positive interaction between peers, family, and within a community [35]. A high risk of alcohol use had a significant association with CMD. This might be due to issues related to alcohol drinking such as financial problem, sickness, job loss, minor conviction, accident, and injury which result in poor economic level and inability to afford health care causing CMD [36, 37].

Having both medical and surgical therapy is significantly associated with the prevalence of CMD this might be related to expenses and to the fear and expectations arising from the combined treatment [38]. Taking systemic carbonic anhydrase inhibitors had a significant association with the prevalence of CMD. Most patients on systemic carbonic anhydrase inhibitors had high intraocular pressure which creates stress and discomfort which give rise to stress and worries [39], these and the drugs’ side effects like fatigue, tingling, anorexia, and weight loss contribute to the development of CMD. This finding was consistent with a study done in Taiwan [40]. Having a chronic medical illness is significantly associated with the prevalence of CMDs. The reason might be those living with chronic medical illness might have limited activity, experience dissatisfaction in life which may expose them to depression and anxiety [8]. This result is consistent with studies done in Ethiopia [8] and Taiwan [40].

Since this study findings showed the prevalence of common mental disorders is associated with different socio-demographic, behavioral and ocular factors it is recommended planning for creating and organizing mental health gap action program training for health professionals working at glaucoma clinic to improve patients’ ocular and mental condition simultaneously. Furthermore, longitudinal studies should be conducted with larger sample size and with other tools that were designed to assess a specific types of psychological disorders.

This study has some limitations. Since cross-sectional study design was used it is difficult to establish a causal relationship between dependent and significant independent variables. The study was conducted in a single institution; it limits its generalizability to general glaucoma patients. Recall bias and social desirability bias were another issues due to the nature of the questionnaires to assess CMD and substance use. In addition, the tool (SRQ -20) used to assess CMD have limitation in screening specific types of CMD.

## Conclusion

The current study revealed a significant number of glaucoma patients have CMD, and it was associated with socio-demographic characteristics (sex and income), level of social support, alcohol use, ocular characteristics (type of treatment, use of systemic carbonic anhydrase inhibitors) and presence of chronic medical illness. Strategies that enable the incorporation of psychosocial care into the current treatment of patients with glaucoma will be beneficial in combating the burden of this comorbidity in glaucoma patients.
